# Phase Retrieval Based on Shaped Incoherent Sources

**DOI:** 10.3390/s23239405

**Published:** 2023-11-25

**Authors:** Ziyan Chen, Jing Cheng, Heng Wu

**Affiliations:** 1 Guangdong Provincial Key Laboratory of Cyber-Physical System, School of Automation, Guangdong University of Technology, Guangzhou 510006, China; zychen@gdut.edu.cn; 2 School of Computer, Guangdong University of Technology, Guangzhou 510006, China; 3 School of Physics, South China University of Technology, Guangzhou 510641, China; phjcheng@scut.edu.cn

**Keywords:** ghost imaging, phase retrieval, Fourier optics, signal processing

## Abstract

Current ghost imaging phase reconstruction schemes require either complex optical systems, iterative algorithms, Fourier transform steps, or entangled photon pairs. These factors may increase the difficulty of system design, lead to phase retrieval errors, or result in excessive time consumption. To tackle this challenge, we propose a five-step phase-shifting method that eliminates the need for complex optical systems, Fourier transform steps, entangled photon pairs, or iterative algorithms. Using five specifically designed incoherent sources, we can generate five distinct ghost imaging patterns. Subsequently, the phase information of the object can be calculated from these five speckle patterns. Additionally, we offer a detailed theoretical explanation for choosing the five-step phase-shifting method over the more commonly used three-step or four-step phase-shifting methods. We demonstrate the applicability of this theoretical proposal through numerical simulations involving two types of complicated objects. The results illustrate that the phase information of the complex object can be successfully and quantitatively reconstructed.

## 1. Introduction

The ghost imaging (GI) technique is a novel imaging method that can non-locally retrieve information about an unknown object through correlation measurements of intensity fluctuations in two detectors. One of these detectors is referred to as the test detector, which typically lacks resolution and is commonly a bucket detector or point detector placed in the test path. The other detector is known as the reference detector and possesses high resolution in the reference path [1,2]. With the development of GI technology, a plethora of other ghost imaging schemes have emerged, such as computational ghost imaging [3,4,5,6,7], differential ghost imaging [8,9,10], corresponding ghost imaging [11,12], normalized ghost imaging [13], Fourier ghost imaging [14,15], and sine ghost imaging [16]. GI technology exhibits outstanding low-light imaging capabilities, enabling the generation of clear images from extremely weak light signals. This capability holds tremendous potential for various applications in scenarios characterized by low signal-to-noise ratios, including medical imaging [17], cryptography [18,19], remote sensing [20,21], and watermarking [22].

Much of the work in GI has traditionally focused on retrieving the amplitude of an unknown object while neglecting its phase. However, in recent years, various methods have been reported for obtaining the phase distributions of unknown objects in GI. In 2006, the first phase retrieval method based on GI was introduced, extracting both amplitude and phase information from the measurement of the first-order spatial correlation function using a modified Young interferometer [23]. Subsequently, in 2008, another GI method based on numerous numerical iteration calculations successfully reconstructed phase distributions [24]. In 2010, Gong demonstrated a modified GI system incorporating two mirrors and two beam splitters (BSs) in the standard GI scheme, enabling the separate and non-local reconstruction of both the complex-valued object and its amplitude-dependent part [25]. In 2012, a ghost hologram was utilized to record with a single-pixel configuration, adapting concepts from standard digital holography to extract the intensity and phase information of a structured and realistic object [26]. In 2014, Zhang reported an experimental realization of lens-less ghost imaging for a phase-only object with pseudo-thermal light. In contrast to conventional ghost imaging, the scheme involves the interference of two correlated fields, and the phase information of the object can be retrieved [27]. In 2018, Sui introduced a novel computational ghost imaging scheme that utilizes specially designed phase-only masks to efficiently encrypt an original image into a series of measured intensities. This method achieves a high-quality reconstruction of the original information through the application of an iterative phase retrieval algorithm [28]. Also in the same year, Sui proposed a single-pixel correlated imaging scheme that employs an iterative phase retrieval algorithm [29]. In 2019, a modified ghost diffraction method combined with the four-step phase-shifting approach was reported to extract the relative phase information of a double-slit with a fixed phase difference between the two slits and a Gaussian phase distribution plate [30,31]. In 2021, Li developed an efficient and robust diffraction imaging scheme based on single-pixel imaging and successfully reconstructed a complex value image using a truncated amplitude flow phase retrieval algorithm [32]. Recently, Sephton disclosed that essential interference required for phase retrieval is inherently present in the correlation measurements derived from conventional projective masks in bi-photon quantum ghost imaging. He devised a straightforward method to acquire comprehensive phase and amplitude data for intricate objects [33].

The aforementioned phase reconstruction approaches can be broadly categorized into four main types. The first type relies on intricate optical path designs, as seen in methods such as [25,27]. Due to the need for complex optical path designs, this category of methods introduces certain challenges in phase reconstruction. The second type is based on Fourier transforms, exemplified via methods like [23,24,26,30,31,34]. While effective, these approaches may introduce some errors in the results due to the necessity of Fourier transformations. The third type is founded on iterative algorithms, as demonstrated in [28,29,32]. The nature of these methods, requiring multiple iterations, also extends the reconstruction time significantly. Lastly, the fourth type is based on entangled photon pairs, as shown in [33]. It is noteworthy that this approach, rooted in quantum entanglement ghost imaging, mandates the use of entangled photon pairs and cannot be accomplished with classical light sources. In this paper, we introduce a five-step phase-shifting method that directly acquires the phase information of complex objects without relying on a complex ghost imaging system, iterative algorithms, Fourier transform steps, or entangled photon pairs. This method addresses the limitations of existing approaches, providing a more efficient and accessible solution to phase retrieval in ghost imaging.

This paper is organized as follows: In Section 2, we present the theoretical model. By designing five different incoherent sources, we can retrieve the phase through direct calculations in ghost imaging (GI). In Section 3, we provide two examples to demonstrate the performance of this phase retrieval proposal. Finally, in Section 4, we conclude the paper.

## 2. Model and Theory

**Figure 1:**
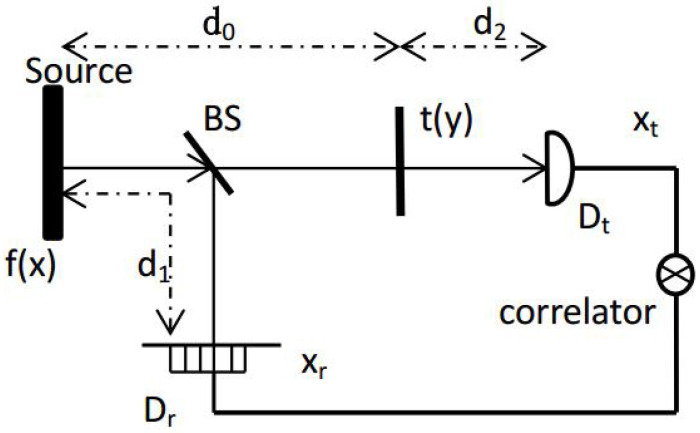
Geometry of the standard GI system. The source field f(x) is split into two beams via the nonpolarized BS. *x*, *y*, $x_{t}$, $x_{r}$ are the positions at the source plane, unknown object plane, test detector plane, and reference detector plane.

Our GI scheme is depicted in Figure 1; it can be observed that it is identical to the standard GI system. The classical stochastic optical field is split into two beams via the nonpolarized beam splitter (BS); these two beams propagate through the reference and test paths. The test path contains an unknown object and a point detector $D_{t}$, with $d_{2}$ representing the distance from the object to the test detector $D_{t}$ and $d_{0}$ representing the distance from the source to the object. The reference path is unrelated to the object and includes a high-resolution detector $D_{r}$, with the distance between the source and the detector $D_{r}$ denoted as $d_{1}$. A correlator correlates the intensity distributions recorded by $D_{t}$ and $D_{r}$ to obtain the GI patterns.

Based on classical optical coherent theory [35] and the observation that the field fluctuations of a classical light source can be modeled via a complex circular Gaussian random process with zero mean [36], we can derive the correlation of the intensity fluctuations between the two detectors

(1) $$\begin{array}{cc} G(x_{t},x_{r})= & \langle E_{r}\left(x_{r}\right)E_{r}^{*}\left(x_{r}\right)E_{t}\left(x_{t}\right)E_{t}^{*}\left(x_{t}\right)\rangle \\ & -\langle E_{t}\left(x_{t}\right)E_{t}^{*}\left(x_{t}\right)\rangle \langle E_{r}\left(x_{r}\right)E_{r}^{*}\left(x_{r}\right)\rangle \end{array}$$

in which $E_{t}\left(x_{t}\right)\left(E_{r}\left(x_{r}\right)\right)$ is the optical field of the test(reference) detector. The field $E_{t}\left(x_{t}\right)$ can be calculated by the Fresnel integral [35]

(2) $$\begin{array}{cc} E_{t}\left(x_{t}\right)= & \frac{-1}{\lambda \sqrt{d_{0}d_{2}}}\int dydx_{1}E_{s}\left(x_{1}\right)\exp\left[-\frac{i\pi }{\lambda d_{0}}{\left(x_{1}-y\right)}^{2}\right]\\ & \times t\left(y\right)\exp[-\frac{i\pi }{\lambda d_{2}}{\left(y-x_{t}\right)}^{2}] \end{array}$$

Then, we can obtain the field $E_{r}\left(x_{r}\right)$ similarly, as follows:

(3) $$\begin{array}{cc} E_{r}\left(x_{r}\right)= & \frac{1}{\sqrt{i\lambda d_{1}}}\int dx_{2}E_{s}\left(x_{2}\right)\exp\left[-\frac{i\pi }{\lambda d_{1}}{\left(x_{2}-x_{r}\right)}^{2}\right] \end{array}$$

where $E_{s}\left(x_{1}\right)\left(E_{s}\left(x_{2}\right)\right)$ is the optical field in the source plane.

Combining Equations (2) and (3) into Equation (1)

(4) $$\begin{array}{cc} G(x_{t},x_{r})= & \frac{1}{\lambda^{3}d_{0}d_{1}d_{2}}|\int dydx_{1}dx_{2}\Gamma \left(x_{1},x_{2}\right)\exp\left[\frac{i\pi }{\lambda d_{1}}{\left(x_{2}-x_{r}\right)}^{2}\right]\\ & \times \exp\left[-\frac{i\pi }{\lambda d_{0}}{\left(x_{1}-y\right)}^{2}\right]t\left(y\right)\exp\left[-\frac{i\pi }{\lambda d_{2}}{\left(y-x_{t}\right)}^{2}\right]{|}^{2} \end{array}$$

in which $\Gamma (x_{1},x_{2})$ represents the coherence function of the source.

Assuming the source is fully spatially incoherent, then we have

(5) $$\Gamma \left(x_{1},x_{2}\right)=\langle E_{s}\left(x_{1}\right)E_{s}^{*}\left(x_{2}\right)\rangle =f\left(x_{1}\right)\delta \left(x_{1}-x_{2}\right)$$

where $f(x_{1})$ is the intensity distribution of the source and 〈〉 represents the ensemble average. Therefore, Equation (4) can be rewritten as

(6) $$\begin{array}{cc} G(x_{t},x_{r})= & \frac{1}{\lambda^{3}d_{0}d_{1}d_{2}}|\int dydxf\left(x\right)\exp\left[\frac{i\pi }{\lambda d_{1}}{\left(x-x_{r}\right)}^{2}\right]\\ & \times \exp\left[-\frac{i\pi }{\lambda d_{0}}{\left(x-y\right)}^{2}\right]t\left(y\right)\exp\left[-\frac{i\pi }{\lambda d_{2}}{\left(y-x_{t}\right)}^{2}\right]{|}^{2} \end{array}$$

To implement GI, the distances are set as $d_{0}=d_{1}$, and a point detector is placed in the test path. Subsequently, we can derive the following GI formula:

(7) $$\begin{array}{ccc} I(x_{r}) & = & G(x_{t}=0,x_{r})\\ & = & \frac{1}{\lambda^{3}d_{0}^{2}d_{2}}|\int f\left(x\right)\exp\left[\frac{i\pi }{\lambda d_{0}}2x\left(y-x_{r}\right)\right]dx\\ & & \times \int t\left(y\right)\exp\left[-\frac{i\pi }{\lambda }\left(\frac{1}{d_{0}}+\frac{1}{d_{2}}\right)y^{2}\right]{dy|}^{2}\\ & = & \frac{1}{\lambda^{3}d_{0}^{2}d_{2}}{\left|\int dyF\left(\frac{x_{r}-y}{\lambda d_{0}}\right)t\left(y\right)\exp\left[-\frac{i\pi }{\lambda }\left(\frac{1}{d_{0}}+\frac{1}{d_{2}}\right)y^{2}\right]\right|}^{2}\\ & = & \frac{1}{\lambda^{3}d_{0}^{2}d_{2}}{\left|F\left(\frac{x_{r}}{\lambda d_{0}}\right)⊗\left(t\left(x_{r}\right)\exp\left[-\frac{i\pi }{\lambda }\left(\frac{1}{d_{0}}+\frac{1}{d_{2}}\right){x_{r}}^{2}\right]\right)\right|}^{2} \end{array}$$

in which *F*(.) is the Fourier transform of *f*(.), and ⊗ denotes convolution.

For convenience, we use object $tt(x_{r})$ to replace $t\left(x_{r}\right)\exp\left[-\frac{i\pi }{\lambda }\left(\frac{1}{d_{0}}+\frac{1}{d_{2}}\right){x_{r}}^{2}\right]$, so we have

(8) $$\begin{array}{c} tt\left(x_{r}\right)=t\left(x_{r}\right)\exp\left[-\frac{i\pi }{\lambda }\left(\frac{1}{d_{0}}+\frac{1}{d_{2}}\right){x_{r}}^{2}\right] \end{array}$$

From Equation (8), we can directly conclude that the amplitude of $tt(x_{r})$ is identical to that of $t(x_{r})$, and their phase difference, which is related to $x_{r}$, is also explicit. Therefore, if we obtain the phase and amplitude information of the object $tt(x_{r})$, we can then reconstruct the phase and amplitude distributions of $t(x_{r})$.

Using Equation (8), we can rewrite Equation (7) as

(9) $$\begin{array}{c} I\left(x_{r}\right)=\frac{1}{\lambda^{3}d_{0}^{2}d_{2}}{\left|F\left(\frac{x_{r}}{\lambda d_{0}}\right)⊗tt\left(x_{r}\right)\right|}^{2} \end{array}$$

We can observe that Equation (9) essentially describes a coherent imaging system, where the object $tt(x_{r})$ undergoes a coherent imaging process with a point spread function (PSF) of $F(\frac{x_{r}}{\lambda d_{0}})$. Consequently, we obtain the corresponding measurable intensity distributions $I(x_{r})$. Mathematically, it is possible to acquire enough incoherent intensity distributions with the assistance of several different PSFs to realize the reconstruction of the phase information of the object $tt(x_{r})$. After several attempts, we determined that five different sources (corresponding to five different PSFs) are both appropriate and necessary to achieve our phase retrieval goal. We design the intensity distributions of the sources as

(10) $$\begin{array}{c} f_{m}\left(x\right)=1+u_{m}e^{\left(i2\pi \varepsilon x\right)}+u_{m}^{*}e^{\left(-i2\pi \varepsilon x\right)} \end{array}$$

where m=1,2,3,4,5, ε is a real constant, and we set $u_{1}=\frac{1}{2},u_{2}=\frac{1+i}{2\sqrt{2}},u_{3}=\frac{i}{2},u_{4}=\frac{-1+i}{2\sqrt{2}},u_{5}=-\frac{1}{2}$ to ensure that $f_{m}\left(x\right)$ is a positive real function. Since $f_{m}\left(x\right)$ represents the intensity distributions of the source, it must be positive and real.

It is evident that we have designed five sets of different light source distributions here. Therefore, why do we need five sets instead of three or four? In other words, why are we using the five-step phase-shifting method here instead of the more commonly used four-step or three-step phase-shifting methods? We will elaborate and provide an explanation for this question in the following. By substituting Equation (10) into Equation (9), we can obtain

(11) $$\begin{array}{c} I\left(x_{r}\right)=\frac{1}{\lambda^{3}d_{0}^{2}d_{2}}{\left|tt\left(x_{r}\right)+u_{m}tt\left(x_{r}-\varepsilon \lambda d_{0}\right)+u_{m}^{*}tt\left(x_{r}+\varepsilon \lambda d_{0}\right)\right|}^{2} \end{array}$$

For convenience, we set $A=\frac{1}{\lambda^{3}d_{0}^{2}d_{2}}$, $\gamma =tt(x_{r})$, $\chi =tt(x_{r}-\varepsilon \lambda d_{0})$, $\nu =tt(x_{r}+\varepsilon \lambda d_{0})$. Then, we can rewrite Equation (11) as

(12) $$\begin{array}{ccc} I(x_{r}) & = & A{\left|\gamma +u_{m}\chi +u_{m}^{*}\nu \right|}^{2}\\ & = & A[|\gamma {|}^{2}+|u_{m}\chi +u_{m}^{*}\nu {|}^{2}+\gamma^{*}\left(u_{m}\chi +u_{m}^{*}\nu \right)+\gamma {\left(u_{m}\chi +u_{m}^{*}\nu \right)}^{*}]\\ & = & A[|\gamma {|}^{2}+|u_{m}\chi {|}^{2}+|u_{m}^{*}\nu {|}^{2}+u_{m}\chi {\left(u_{m}^{*}\nu \right)}^{*}+{\left(u_{m}\chi \right)}^{*}u_{m}^{*}\nu \\ & & +\gamma^{*}\left(u_{m}\chi +u_{m}^{*}\nu \right)+\gamma {\left(u_{m}\chi +u_{m}^{*}\nu \right)}^{*}]\\ & = & A\{\gamma^{2}+{\left(u_{m}\chi \right)}^{2}+{\left(u_{m}^{*}\nu \right)}^{2}+u_{m}^{2}\left[\chi \nu^{*}+{\left(\chi \nu^{*}\right)}^{*}\right]\\ & & +u_{m}\left(\gamma^{*}\chi +\gamma \nu^{*}\right)+u_{m}^{*}{\left(\gamma^{*}\chi +\gamma \nu^{*}\right)}^{*}\} \end{array}$$

To make the above formula look more concise and easy to understand, we once again set $\alpha =\gamma^{2}+{\left(u_{m}\chi \right)}^{2}+{\left(u_{m}^{*}\nu \right)}^{2}$, $\beta =\chi \nu^{*}$, $\omega =\gamma^{*}\chi +\gamma \nu^{*}$; then, Equation (12) can be rewritten as

(13) $$\begin{array}{ccc} I(x_{r}) & = & A(\alpha +u_{m}^{2}\beta +u_{m}^{2}\beta^{*}+u_{m}\omega +u_{m}^{*}\omega^{*}) \end{array}$$

It is evident that α, β, $\beta^{*}$, ω, and $\omega^{*}$ represent the five unknowns in Equation (13). The most essential solution we require is for $\beta =\chi \nu^{*}=tt\left(x_{r}-\varepsilon \lambda d_{0}\right)tt^{*}\left(x_{r}+\varepsilon \lambda d_{0}\right)$. Since Equation (13) involves five unknowns, we need a minimum of five sets of equations to solve for these variables. In other words, neither the three-step nor the four-step phase-shift method can be employed in our proposed approach. This is also the fundamental reason why we employ the five-step phase-shift method in our proposed scheme.

Therefore, with those five sources, we obtain the five corresponding GI patterns as follows:

(14) $$\begin{array}{ccc} & & I_{1}\left(x_{r}\right)=\frac{1}{\lambda^{3}d_{0}^{2}d_{2}}{\left|tt\left(x_{r}\right)+\frac{1}{2}tt\left(x_{r}-\varepsilon \lambda d_{0}\right)+\frac{1}{2}tt\left(x_{r}+\varepsilon \lambda d_{0}\right)\right|}^{2}\\ & & I_{2}\left(x_{r}\right)=\frac{1}{\lambda^{3}d_{0}^{2}d_{2}}{\left|tt\left(x_{r}\right)+\frac{1+i}{2\sqrt{2}}tt\left(x_{r}-\varepsilon \lambda d_{0}\right)+\frac{1-i}{2\sqrt{2}}tt\left(x_{r}+\varepsilon \lambda d_{0}\right)\right|}^{2}\\ & & I_{3}\left(x_{r}\right)=\frac{1}{\lambda^{3}d_{0}^{2}d_{2}}{\left|tt\left(x_{r}\right)+\frac{i}{2}tt\left(x_{r}-\varepsilon \lambda d_{0}\right)-\frac{i}{2}tt\left(x_{r}+\varepsilon \lambda d_{0}\right)\right|}^{2}\\ & & I_{4}\left(x_{r}\right)=\frac{1}{\lambda^{3}d_{0}^{2}d_{2}}{\left|tt\left(x_{r}\right)+\frac{-1+i}{2\sqrt{2}}tt\left(x_{r}-\varepsilon \lambda d_{0}\right)-\frac{1+i}{2\sqrt{2}}tt\left(x_{r}+\varepsilon \lambda d_{0}\right)\right|}^{2}\\ & & I_{5}\left(x_{r}\right)=\frac{1}{\lambda^{3}d_{0}^{2}d_{2}}{\left|tt\left(x_{r}\right)-\frac{1}{2}tt\left(x_{r}-\varepsilon \lambda d_{0}\right)-\frac{1}{2}tt\left(x_{r}+\varepsilon \lambda d_{0}\right)\right|}^{2} \end{array}$$

Then, we solve for the solution of $tt\left(x_{r}-\varepsilon \lambda d_{0}\right)tt^{*}\left(x_{r}+\varepsilon \lambda d_{0}\right)$ using the five sets of equations in Equation (14), resulting in

(15) $$\begin{array}{ccc} \beta (x_{r}) & = & tt\left(x_{r}-\varepsilon \lambda d_{0}\right)tt^{*}\left(x_{r}+\varepsilon \lambda d_{0}\right)\\ & = & \frac{\left(2\sqrt{2}-2\right)\left(1+i\right)}{2\sqrt{2}-4}I_{1}\left(x_{r}\right)+\frac{\left[-2\sqrt{2}+i\left(2\sqrt{2}-4\right)\right]}{2\sqrt{2}-4}I_{2}\left(x_{r}\right)\\ & & +\frac{4}{2\sqrt{2}-4}I_{3}\left(x_{r}\right)+\frac{\left[-2\sqrt{2}-i\left(2\sqrt{2}-4\right)\right]}{2\sqrt{2}-4}I_{4}\left(x_{r}\right)\\ & & +\frac{\left(2\sqrt{2}-2\right)\left(1-i\right)}{2\sqrt{2}-4}I_{5}\left(x_{r}\right) \end{array}$$

It is natural to obtain

(16) $$\begin{array}{c} \Phi_{\beta }\left(x_{r}\right)=\Phi_{tt}\left(x_{r}-\varepsilon \lambda d_{0}\right)-\Phi_{tt}\left(x_{r}+\varepsilon \lambda d_{0}\right) \end{array}$$

where $\Phi_{\beta }\left(x_{r}\right)$ ($\Phi_{tt}\left(x_{r}\right)$) is the phase of $\beta (x_{r})$ ($tt(x_{r})$). One can assume that the phase at tt=0 is zero, i.e., $\Phi_{tt}\left(0\right)=0$; therefore, this equation can give the values of $\Phi_{tt}\left(0\right)$, $\pm \Phi_{tt}\left(2\varepsilon \lambda d_{0}\right)$, $\pm \Phi_{tt}\left(\varepsilon \lambda d_{0}\right)$,… Now, it is evident that we have successfully reconstructed the phase of $tt(x_{r})$ with the phase information of $\beta (x_{r})$. Therefore, the next step is to obtain the phase distributions of $t(x_{r})$ (this is exactly what we need to obtain in our scheme) from the phase values of $tt(x_{r})$, and with Equation (8) we can directly obtain

(17) $$\begin{array}{c} \Phi_{t}\left(x_{r}\right)=\Phi_{tt}\left(x_{r}\right)+\frac{i\pi }{\lambda }\left(\frac{1}{d_{0}}+\frac{1}{d_{2}}\right){x_{r}}^{2} \end{array}$$

in which $\Phi_{t}\left(x_{r}\right)$ represents the phase of $t(x_{r})$. Consequently, using Equation (17) and the values of $\Phi_{tt}\left(0\right)$, $\pm \Phi_{tt}\left(2\varepsilon \lambda d_{0}\right)$, $\pm \Phi_{tt}\left(4\varepsilon \lambda d_{0}\right)$, and so on, one can directly obtain the values of $\Phi_{t}\left(0\right)$, $\pm \Phi_{t}\left(2\varepsilon \lambda d_{0}\right)$, $\pm \Phi_{t}\left(4\varepsilon \lambda d_{0}\right)$, and so forth. It is noteworthy that we have directly acquired the phase of the object $t(x_{r})$ without the need for the Fourier transform step. In contrast, in the work [30,31], it is imperative to employ the Fourier transform step to reconstruct the phase information of the unknown object. Since we have assumed that the phase at $tt(x_{r})=0$ is zero, we can state that $\phi_{tt}\left(0\right)=0$. Consequently, the reconstructed phase will have a constant value difference from the real phase. However, this absolute phase is not significant because the relative phase distribution remains invariant.

## 3. Numerical Simulations

In the following discussions, we select two different types of complicated objects (four-slit and double-slit Gaussian plates) to validate the effectiveness of our GI scheme. In our simulations, we set the source’s transverse size $D_{s}$ to be 10 mm, the wavelength λ to be 628 nm, the distance between the source and the object in the test path to be $d_{0}$ = 400 mm, and the distance between the object and the test detector $D_{t}$ to be $d_{2}$ = 200 mm. The resolution of the CCD is $\Delta_{x_{r}}$ = 8.3 μm, the sample number is M = 320, and we set ε = 3.3041 × 10${}^{-5}$ nm${}^{-1}$.

**Figure 2:**
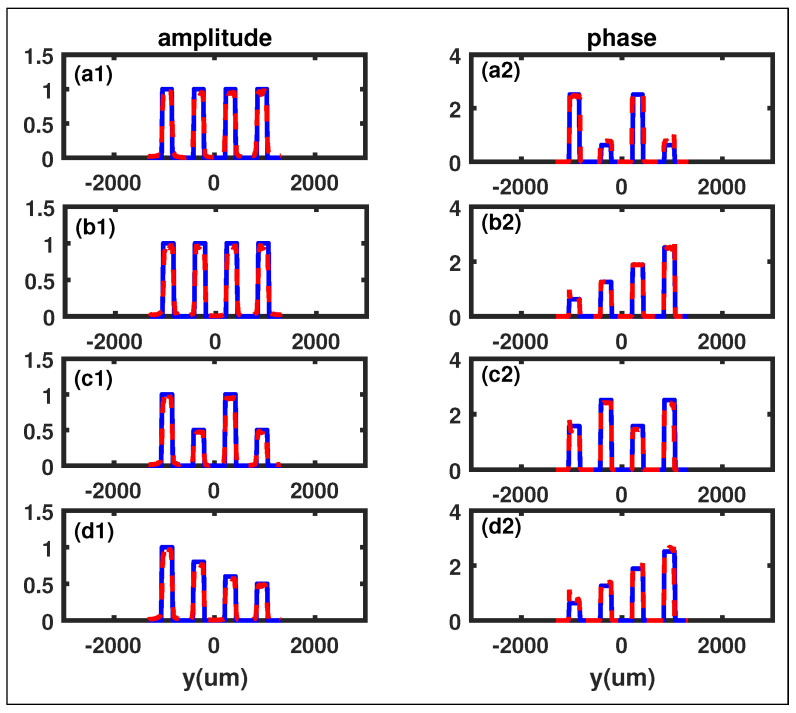
The retrieved phases and amplitudes of the four different four-slits. The solid blue curves represent the initial phases and amplitudes, while the dashed red curves display the reconstructed phases and amplitudes. (**a1**,**a2**): The original amplitude and phase of the first four-slit, with parameters $\alpha_{1}=\alpha_{2}=\alpha_{3}=\alpha_{4}=1$, $\theta_{\alpha 1}=\theta_{\alpha 3}=0.8\pi$, $\theta_{\alpha 2}=\theta_{\alpha 4}=0.2\pi$. (**b1**,**b2**): The original amplitude and phase of the second four-slit, with parameters $\alpha_{1}=\alpha_{2}=\alpha_{3}=\alpha_{4}=1$, $\theta_{\alpha 1}=0.2\pi ,\theta_{\alpha 2}=0.4\pi$, $\theta_{\alpha 3}=0.6\pi ,\theta_{\alpha 4}=0.8\pi$. (**c1**,**c2**): The original amplitude and phase of the third four-slit, with parameters $\alpha_{1}=\alpha_{3}=1,\alpha_{2}=\alpha_{4}=0.5$, $\theta_{\alpha 1}=\theta_{\alpha 3}=0.5\pi$, $\theta_{\alpha 2}=\theta_{\alpha 4}=0.8\pi$. (**d1**,**d2**): The original amplitude and phase of the fourth four-slit, with parameters $\alpha_{1}=1,\alpha_{2}=0.8,\alpha_{3}=0.6,\alpha_{4}=0.5$, $\theta_{\alpha 1}=0.2\pi ,\theta_{\alpha 2}=0.4\pi$, $\theta_{\alpha 3}=0.6\pi ,\theta_{\alpha 4}=0.8\pi$.

The transmittance of the four-slit is given by

$$t_{1}\left(y\right)=\left\{\begin{array}{ccc} \alpha_{1}e^{i\theta_{\alpha 1}} & \; & -\frac{4w+3d}{2}\leq y\leq -\frac{2w+3d}{2}\\ \alpha_{2}e^{i\theta_{\alpha 2}} & \; & -\frac{2w+d}{2}\leq y\leq -\frac{d}{2}\\ \alpha_{3}e^{i\theta_{\alpha 3}} & \; & \frac{d}{2}\leq y\leq \frac{2w+d}{2}\\ \alpha_{4}e^{i\theta_{\alpha 4}} & \; & \frac{2w+3d}{2}\leq y\leq \frac{4w+3d}{2}\\ 0 & \; & \mathrm{other} \end{array}\right.$$

where *w* represents the slit width, *d* represents the slit distance, $\alpha_{i}$ and $\theta_{\alpha i}$ are the amplitude and phase of the $i_{th}$ slit, respectively. We set w=210μm and d=420μm. Here, we choose four different four-slits to demonstrate the dependability of our GI method. The reconstructed phases and amplitudes of the four-slits have been displayed in Figure 2. The first four-slit, shown in Figure 2(a1,a2), has parameters $\alpha_{1}=\alpha_{2}=\alpha_{3}=\alpha_{4}=1$, $\theta_{\alpha 1}=\theta_{\alpha 3}=0.8\pi$, $\theta_{\alpha 2}=\theta_{\alpha 4}=0.2\pi$. It has the same amplitude in every slit, and its retrieval phase and amplitude results are successfully reconstructed as expected. The second four-slit, shown in Figure 2(b1,b2), has parameters $\alpha_{1}=\alpha_{2}=\alpha_{3}=\alpha_{4}=1$, $\theta_{\alpha 1}=0.2\pi ,\theta_{\alpha 2}=0.4\pi$, $\theta_{\alpha 3}=0.6\pi ,\theta_{\alpha 4}=0.8\pi$. Its amplitude is the same as the first four-slit, but its phase distributions in every slit are different from each other. The third four-slit, shown in Figure 2(c1,c2), has parameters $\alpha_{1}=\alpha_{3}=1,\alpha_{2}=\alpha_{4}=0.5$, $\theta_{\alpha 1}=\theta_{\alpha 3}=0.5\pi$, $\theta_{\alpha 2}=\theta_{\alpha 4}=0.8\pi$. Its amplitude and phase have also been successfully reconstructed. The fourth four-slit, shown in Figure 2(d1,d2), is very complicated, as its phase and amplitude are different in every slit. Its parameters are $\alpha_{1}=1,\alpha_{2}=0.8,\alpha_{3}=0.6,\alpha_{4}=0.5$, $\theta_{\alpha 1}=0.2\pi ,\theta_{\alpha 2}=0.4\pi$, $\theta_{\alpha 3}=0.6\pi ,\theta_{\alpha 4}=0.8\pi$, and it has also been successfully reconstructed under our GI scheme.

The relative difference between the reconstructed and the original phases is near zero, consistent with our theory demonstrated in Section 2. To further verify our GI scheme, we have also simulated another more complicated object (double-slit Gaussian plate) than the four-slits for the second example.

**Figure 3:**
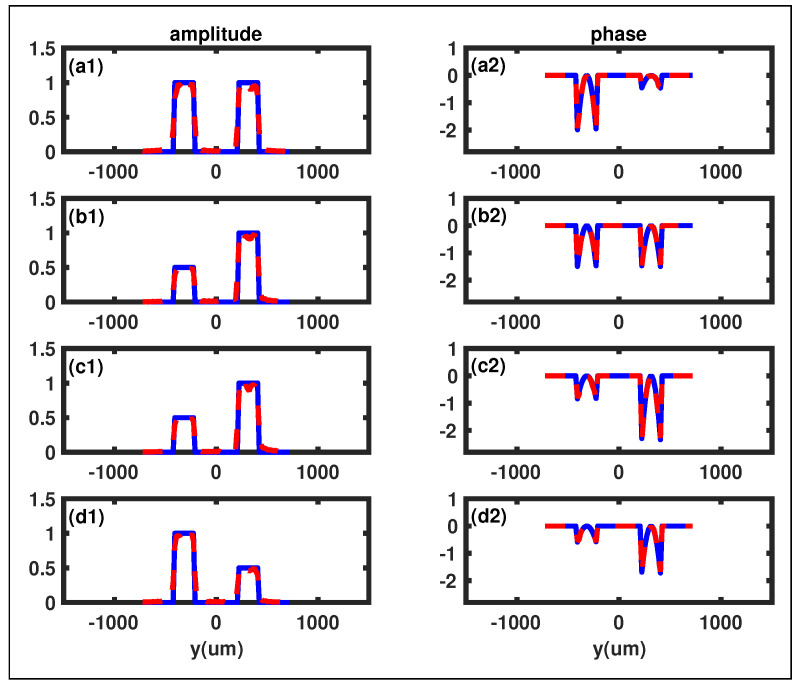
The retrieved phases and amplitudes of the four different double-slit Gaussian plates. The solid blue curves represent the initial phases and amplitudes, while the dashed red curves display the reconstructed phases and amplitudes. (**a1**,**a2**): The original amplitude and phase of the first double-slit Gaussian plate, with parameters $\beta_{1}=\beta_{2}=1$, $b_{1}=65\;\mu$m, $b_{2}=135\;\mu$m. (**b1**,**b2**): The original amplitude and phase of the second double-slit Gaussian plate, with parameters $\beta_{1}=0.5$, $\beta_{2}=1$, $b_{1}=b_{2}=75\;\mu$m. (**c1**,**c2**): The original amplitude and phase of the third double-slit Gaussian plate, with parameters $\beta_{1}=0.5$, $\beta_{2}=1$, $b_{1}=100\;\mu$m, $b_{2}=60\;\mu$m. (**d1**,**d2**): The original amplitude and phase of the fourth double-slit Gaussian plate, with parameters $\beta_{1}=1$, $\beta_{2}=0.5$, $b_{1}=120\;\mu$m, $b_{2}=70\;\mu$m.

The second example, as shown in Figure 3, is a double-slit Gaussian plate represented by

$$t_{2}\left(y\right)=\left\{\begin{array}{ccc} \beta_{1}e^{-\frac{iy^{2}}{b_{1}^{2}}} & \; & -\frac{2w+d}{2}\leq y\leq -\frac{d}{2}\\ \beta_{2}e^{-\frac{iy^{2}}{b_{2}^{2}}} & \; & \frac{d}{2}\leq y\leq \frac{2w+d}{2}\\ 0 & \; & \mathrm{other} \end{array}\right.$$

in which $\beta_{i}$ is the amplitude of the $i_{th}$ Gaussian plate slit, and $b_{i}$ represents the width parameter of the phase distribution for the $i_{th}$ Gaussian plate slit.

It is evident from Figure 3 that both amplitudes and phases of the double-slit Gaussian plate have been successfully reconstructed. The relative difference between the reconstructed phases and the initial phases is also near zero, further demonstrating the validity of our GI phase retrieval scheme.

## 4. Conclusions

In summary, we present an algorithm known as the five-step phase-shifting method for reconstructing the phase distribution of an unknown object based on ghost imaging. This method does not require any complex ghost imaging systems, iterative algorithms, Fourier transform steps, or entangled photon pairs. To demonstrate the effectiveness of our ghost imaging scheme, we simulated two types of complex objects (four-slit and double-slit Gaussian plates), indicating the reliability of our approach.

Moreover, while our method was initially designed for dual-arm ghost imaging, it seamlessly extends to single-arm ghost imaging, commonly known as computational ghost imaging. In contrast to the previous approach [30,31], there are two advantages to the current five-step phase-shifting method. First, the current method no longer requires any Fourier transforms. Second, the previous method could only be used with dual-arm ghost imaging because it required placing a reference screen in the reference arm, making it impossible to obtain the light field distribution of the reference optical path through computation. In our current approach, the reference path does not contain any objects like a reference screen, allowing for the direct calculation of the light field distribution of the reference optical path. This adaptability enhances the practical utility of our method, making it applicable to computational ghost imaging scenarios.

Lastly, we would like to emphasize that, while our theoretical derivation is based on one-dimensional calculations, our method can equally be applied to two-dimensional optical fields. We plan to explore the application of our approach to two-dimensional optical fields in future research.

## Data Availability

Data underlying the results presented in this paper are not publicly available at this time but may be obtained from the authors upon reasonable request.
